# Influence of Hurdle Technology on Foodborne Pathogen Survival in the Human Gastrointestinal Tract

**DOI:** 10.3390/microorganisms11020405

**Published:** 2023-02-06

**Authors:** Theodora Akritidou, Simen Akkermans, Cindy Smet, Fien de Mey, Jan F. M. Van Impe

**Affiliations:** BioTeC+, Chemical and Biochemical Process Technology and Control, Department of Chemical Engineering, KU Leuven, 9000 Ghent, Belgium

**Keywords:** hurdle concept, *in vitro* digestion, stress adaptation, *Salmonella* Typhimurium, *Listeria monocytogenes*

## Abstract

The application of several sublethal stresses in hurdle technology can exert microbial stress resistance, which, in turn, might enable foodborne pathogens to overcome other types of lethal stresses, such as the gastrointestinal barriers. The present study evaluated the survival of *Salmonella* Typhimurium and *Listeria monocytogenes* during simulated digestion, following exposure to combinations of water activity (*a*_w_), pH and storage temperature stresses. The results revealed that both pathogens survived their passage through the simulated gastrointestinal tract (GIT) with their previous habituation to certain hurdle combinations inducing stress tolerance. More specifically, the habituation to a low temperature or to a high pH resulted in the increased stress tolerance of *Salmonella*, while for *Listeria*, the cells appeared stress tolerant after exposure to a high temperature or to a low pH. Nonetheless, both pathogens expressed increased sensitivity after habituation to growth-limiting hurdle combinations. The survival of stress-tolerant pathogenic cells in the human GIT poses major public health issues, since it can lead to host infection. Consequently, further research is required to obtain a deeper understanding of the adaptive stress responses of foodborne bacteria after exposure to combinations of sublethal hurdles to improve the existing food safety systems.

## 1. Introduction

The food manufacturing sector has employed a number of practices and resources to improve and maintain food safety. Yet, in 2019, more than 300,000 confirmed cases of foodborne illnesses have been reported in the EU, with *Salmonella enterica* and *Listeria monocytogenes* occurring among the most frequent agents for human infections [1]. In addition, the recent consumer demands for minimally processed, fresh-like and more natural foods, along with their increased awareness about food safety, put further pressure on the present food safety management systems. A promising response to these concurrent consumer requests has been the application of the so-called hurdle technology in food processing [2,3].

The term “hurdle technology” is used to describe the application of more than one preservative treatment at a low dosage, which are often called hurdles, that extend product shelf-life without nullifying its nutritional and sensory value [4]. The mode of action of the hurdle technology relies on combinations of hurdles, with synergistic or additive effects, aiming at the inhibition or inactivation of targeted microorganisms [2]. In the past, hurdle technology was largely used in an empirical manner, considering the lack of profound knowledge regarding its governing principles. Nevertheless, nowadays, the intelligent application of the hurdle concept is highly prevalent in the food industry, since a deeper understanding of the intrinsic and extrinsic food factors has been established. In the last decades, the influence of temperature, water activity (*a*_w_), pH, redox potential (Eh) and competitive microflora, along with their interactions on pathogenic microbial behavior and physiology in food, have been widely studied, amplifying the intelligent use of hurdles in food manufacturing [5].

Interestingly, despite all the advantages of hurdle technology, there is the potential for foodborne pathogens to benefit from this food preservation method rather than being harmed. In view of the sub-inhibitory levels in which each of the combined hurdles is applied, microbial adaptive stress responses may be triggered that could permit the survival or even growth of a significant fraction of the microbial population [6]. This phenomenon describing the ability of a pathogen to respond to harsh environments has often been characterized as induced tolerance, stress adaptation or “stress hardening” [7]. Bacterial survival and tolerance in stressful conditions are usually dictated by the microorganism’s physiological responses, such as modifications occurring in the cell concerning protein expression and activity, and alterations in the cell membrane and morphology. Furthermore, these bacterial physiological responses could lead to cross-protection of the pathogen toward other environmental stresses, inducing increased microbial persistence [6]. An example of these bacterial adaptive stress responses that is related to microbial protection is the acid tolerance response (ATR) mechanism, which involves a number of metabolic and regulatory processes that enable bacteria to survive otherwise lethal stresses. Its activation depends on the previous exposure of a microbe to a mild stress [8].

The impact of bacterial stress on the stress tolerance and persistence of foodborne pathogens is a major concern for food safety management, given that stress adaptation of foodborne pathogens may lead to critical implications for public health, such as the prolonged survival of pathogens throughout the food chain. In addition, pathogen survival in the human gastrointestinal tract (GIT) during digestion can be also a catastrophic outcome of microbial stress adaptation, leading to host infection [9]. The human gut employs a number of antimicrobial mechanisms to impede pathogen colonization. Upon ingestion, a pathogen needs to overcome an assortment of formidable barriers before reaching an optimal environmental niche to adhere and colonize [10,11]. Gastric acidity is often characterized as the first line of defense against foodborne pathogens. The gastricidal action originates from its low pH values, which in the fasted stomach state range from pH 1.0 to 3.0 [12,13]. In addition, an arsenal of other barriers succeeds the stomach in the small intestine, including the detergent properties of bile acids, the limited oxygen availability, the antimicrobial substances secreted by the intestinal epithelium and gut microbiota. Overcoming all these antimicrobial strategies of the human GIT is the key for pathogen colonization in the gut and, ultimately, for host infection [11,14].

This paper aims to investigate the impact of the hurdle technology on the survival patterns of *S*. Typhimurium and *L. monocytogenes* in the upper human GIT. To achieve this goal, a standard meal was contaminated with either of the two pathogens, whose microbial dynamics were monitored throughout the whole simulated digestion. Different hurdle combinations of *a*_w_, pH and storage temperature were applied for the preparation and storage of this meal, facilitating the simulation of food processing conditions. Then, after a storage period of six days under these conditions, the contaminated standard meal was subdued to an *in vitro* digestion process, representing the oral, gastric and proximal small intestinal (duodenum and jejunum) digestion phases.

## 2. Materials and Methods

### 2.1. Microorganisms and Preculture Conditions

*Salmonella enterica* serovar Typhimurium LMG 14933 (isolated from bovine liver) and *Listeria monocytogenes* strain LMG23775 (isolated from sausages) were obtained from the BCCM/LMG bacteria collection of Ghent University in Belgium. The food origin of the bacterial strains dictated their selection. The stock cultures of *S*. Typhimurium were stored frozen (−80 °C) in Tryptone Soy Broth (TSB (Oxoid Ltd., Basingstoke, UK)) with 20% *v*/*v* glycerol (Acros Organics, Morris Plains, NJ, USA), whilst the stock cultures of *L. monocytogenes* were stored similarly, but in Brain–Heart Infusion (BHI) broth (Oxoid Ltd., Basingstoke, UK). With respect to the working cultures, they were renewed every month and they were stored at 5 °C on Tryptone Soy Agar plates (TSA (Oxoid Ltd., Basingstoke, UK)) for *S*. Typhimurium and on BHI agar plates for *L. monocytogenes*. The preparation of the precultures was conducted by transferring one colony from the working cultures to a 50 mL Erlenmeyer flask containing 20 mL of TSB for *S*. Typhimurium and 20 mL of BHI broth for *L. monocytogenes*. Then, the flasks were incubated at 37 °C for 24 h until the cells reached the late stationary phase.

### 2.2. Inoculum Preparation

For the preparation of the inoculum, an aliquot of the preculture of each microorganism was used to inoculate the food model system at about 10^9^ CFU (or 20.7 ln(CFU)). For each experiment, a calculation of the exact volume of this aliquot was implemented, which was based on a 1:10 dilution of the preculture optical density measurement at 595 nm (reference absorbances at 595 nm was 0.09 for *S*. Typhimurium and 0.19 for *L. monocytogenes*).

### 2.3. Food Model System Development and Preparation

A food model system (FMS) based on a standard diet was developed, with an energy (E%) intake of 15% proteins, 35% fat and 50% carbohydrates. The composition of the FMS was 4.2% *w*/*w* whey protein (Power Supplements BV, NL) as a source of proteins, 4.3% *w*/*w* corn oil (Delhaize proxy, Ghent, Belgium) as a source of fat, 13.9% *w*/*w* soluble potato starch (Alfa Aesar, Haverhill, MA, USA) as a source of carbohydrates, NaCl (Sigma-Aldrich, St. Louis, MO, USA) and water. The total amount of the FMS was 100 g, and the salt concentration varied to obtain the desired water activity (*a*_w_). In addition, the pH of the FMS was adjusted according to this research’s demands. All FMSs were constructed as sterile oil-in-water emulsions [15,16], opting for stable and homogeneous food matrixes. In addition, all FMSs were supplemented with nonionic surfactants, i.e., Tween 80 (Sigma-Aldrich, St. Louis, MO, USA) and Span 80 (Sigma-Aldrich, St. Louis, MO, USA). The storage of each FMS was at 37 °C to preserve the solution’s viscosity, and 100 g of FMS was transferred aseptically in an empty sterile 1 L Schott bottle the day of the experiment.

### 2.4. Assessment and Modelling of Boundaries of Growth

As a preliminary task of this study, the growth limits of *S*. Typhimurium and *L. monocytogenes* were under investigation in order to select the appropriate hurdles to be applied for the preparation and storage of the FMS. A full factorial experimental design was followed, where a total of 84 combinations of temperature (10 °C, 25 °C), pH (3.8, 4.2, 4.6, 5.0, 5.6, 5.9, 6.6) and *a*_w_ (0.909, 0.931, 0.949, 0.957, 0.982, 0.994), in four replicates for each combination, were examined for both microorganisms by using the FMS as the basal medium. After autoclaving the FMS, the pH was adjusted aseptically to the desired values using 3 N HCl (ThermoFisher Scientific, Waltham, MA, USA) or 1 N NaOH (VWR, Radnor, PA, USA). With respect to *a*_w_, NaCl was used to attain the appropriate values, which were measured with a water activity meter (AWK-40, Nagy, Gäufelden, Germany) after autoclaving the FMS. The amount of NaCl added in the FMS was decided based on a calibration curve of *a*_w_ versus NaCl concentration (conducted in the FMS) that was acquired by a preliminary experiment. Sterile microcentrifuge vials containing 290 μL of the appropriate FMS for each combination were inoculated with 10 μL of late stationary phase cells of *S*. Typhimurium or *L. monocytogenes*. The targeted inoculum size was 10^7^ CFU/ mL. Then, the vials were covered with parafilm to avoid dehydration and stored at 10 °C or 25 °C for 6 days, with the storage time accounting for food products with a shelf-life of one week. For the determination of the actual inoculum level of each microorganism, 24 microcentrifuge vials were sampled immediately after inoculation, and three drops of 20 μL of the appropriate serial decimal dilution in 0.85% *w*/*v* NaCl solution were inoculated on TSA (*S*. Typhimurium) or BHI agar (*L. monocytogenes*) petri dishes. The plates were then incubated at 37 °C for 24 h, and the inoculum level was determined by the enumeration of the colonies. The assessment of growth in the different combinations of FMS was tested after 6 days of storage by inoculating three drops of 20 μL of the appropriate serial decimal dilution on solid general media, as described above. The occurrence of growth was confirmed when the cell density of the microorganism was higher than the average initial cell density plus three times its standard deviation.

A mathematical model was used to illustrate the boundary between the experimentally determined growth and no growth conditions. The four datasets for each combination of temperature and pathogen were fitted with a separate logistic regression model that included linear effects and multiplicative interactions between pH and water activity.

### 2.5. Hurdle Technology Application on the Developed FMS

After the determination of the growth/no growth boundaries of *S*. Typhimurium and *L. monocytogenes*, a two-level factorial design consisting of eight different combinations of temperature, pH and *a*_w_ were selected for the preparation and storage of the developed FMS. These combinations were designated in such a way that four conditions allowed microbial growth and four conditions inhibited microbial growth (survival) for both microorganisms.

### 2.6. Enzymes and Bile Acids

The enzymes used during the *in vitro* digestion process were all purchased from Sigma-Aldrich (St. Louis, MO, USA) and were α-amylase from hog pancreas (≥50 units/mg protein) for the oral phase, pepsin from porcine gastric mucosa (≥400 units/mg protein) for the gastric phase, and pancreatin from porcine and pancreas (8× USP) for the intestinal phase. Furthermore, bile acids were provided during the intestinal phase with the use of porcine bile extract (Sigma-Aldrich, St. Louis, MO, USA).

### 2.7. Simulated Digestion Fluids

Three simulated digestion fluids were used during the *in vitro* digestion process, one for each phase of digestion: the Simulated Salivary Fluid (SSF), the Simulated Gastric Fluid (SGF) and the Simulated Intestinal Fluid (SIF). Their composition was based on the protocol of INFOGEST [17], and each solution contained the necessary amounts of the following stock solutions: 0.50 M KCl (VWR, PA, USA), 0.50 M KH_2_PO_4_ (ThermoFisher Scientific, MA, USA), 1.00 M NaHCO_3_ (Carl Roth, Karlsruhe, Germany), 2.00 M NaCl, 0.15 M MgCl_2_(H_2_O) (Carl Roth, Karlsruhe, Germany), 0.50 M (NH_4_)_2_CO_3_ (Carl Roth, Karlsruhe, Germany) and 0.30 M CaCl_2_(H_2_O)_2_ (VWR, PA, USA). The latter was added individually in the digesta, as precipitation occurred when it was added in the simulated digestion stock solutions. In addition, given that during the digestion process enzymes, calcium chloride and water were added as well, the simulated digestion fluids were prepared at 1.25× concentration in order to obtain the desired ionic composition in the final digestion mixture. The simulated digestion fluids were autoclaved at 121 °C for 15 min along with the calcium chloride solution and the water. The enzyme solutions were prepared by mixing the enzyme powder aseptically in sterile water. All the solutions used during the *in vitro* digestion process were stored refrigerated and were prewarmed at 37 °C overnight, apart from the enzyme solutions that were freshly prepared. Furthermore, in order to improve pancreatin solubility, the solution was freshly prepared and stirred at 400 rpm on ice for 2 h.

### 2.8. In Vitro Digestion Model System

**Figure 1:**
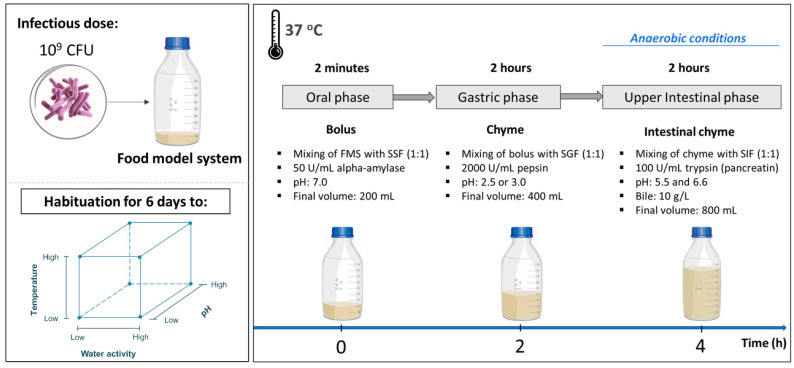
Schematic overview of the implemented experimental protocol.

The INFOGEST static *in vitro* digestion standardized protocol was used to mimic the human digestion in the upper GIT after applying some minor adaptations [17]. A schematic representation of this study’s experimental process is demonstrated in Figure 1. The simulated digestion process consisted of an oral, a gastric and an intestinal phase, with the latter corresponding to the proximal small intestinal segments, duodenum and jejunum. Temperature control was achieved by placing the digestion set-up in an incubator at 37 °C, and mixing of the digesta was conducted by stirring at 400 rpm. The control of pH was accomplished with the addition of the appropriate amounts of 3 M HCl (ThermoFisher Scientific, MA, USA) or 1 M NaOH (VWR, PA, USA), and their required volumes were pre-determined for each step of digestion in a test prior to digestion experiments. Briefly, during the oral phase, the developed FMS was mixed with SSF at a ratio of 1:1 (*w*/*w*), including 500 μL of 0.3 M CaCl_2_(H_2_O)_2_, the enzyme solution and sterile water, to achieve a 1× concentration of the SSF. In fact, after the pH was adjusted to 7.0, 10 mL of a fresh 3% *w*/*v* α-amylase solution was added, aiming to achieve an enzymatic activity of 75 U/mL in the final mixture. The duration of the oral digestion process was 2 min. Afterwards, the oral bolus was mixed with SGF at a ratio of 1:1 (vol/vol) that included 100 μL of 0.3 M CaCl_2_(H_2_O)_2_, the enzyme solution, HCl and sterile water, marking the beginning of the gastric phase. In specific, 10 mL of a 20% *w*/*v* porcine pepsin solution was added in order to achieve a final enzymatic activity of 2000 U/mL in the gastric chyme. After the addition of pepsin, the pH of the gastric phase was adjusted to 2.5 for the digestion experiments focusing on *S*. Typhimurium and to pH 3.0 for *L. monocytogenes*. The difference in the gastric acidity when investigating the behavior of the two pathogens can be explained by the need to apply similar microbial acidic shock and the difference in pH resistance of the two microorganisms as established in an earlier study [16]. The duration of the gastric phase was 2 h, and it was followed by the intestinal phase, in the beginning of which the gastric chyme was mixed with SIF at a ratio of 1:1 (vol/vol). Then, the digesta was further supplemented with 800 μL of 0.3 M CaCl_2_(H_2_O)_2_, 50 mL of a 16% *w*/*v* porcine bile extract solution (10 g/L in the final mixture), 8 mL of a 10% *w*/*v* L-cysteine hydrochloride monohydrate solution (Alfa Aesar, MA, USA), 800 μL of a 50% *w*/*v* resazurin solution (Alfa Aesar, MA, USA) and sterile water (attaining a 1× concentration of the SIF). The addition of L-cysteine hydrochloride and resazurin served for the reduction in the redox potential in the mixture and for monitoring the changes in the redox potential, respectively [18,19]. With respect to the intestinal pH, during the first hour of intestinal digestion, it was adjusted to 5.5, mimicking as such the duodenal acidic conditions, and during the second hour of intestinal digestion, it was fixed to 6.6, simulating the jejunal acidic conditions. After the pH adjustment, 100 mL of a 0.38% *w*/*v* pancreatin solution was added to attain a final trypsin activity of 100 U/mL. Lastly, the digesta was flushed with anoxic nitrogen gas for the whole duration of the intestinal phase, given the necessity for anaerobic conditions during the intestinal phase.

### 2.9. Hurdle Technology Application and Microbiological Analysis

As an initial step, each FMS with a specific pH–*a*_w_ combination was inoculated with an infectious dose of 10^9^ CFU of either *S*. Typhimurium or *L. monocytogenes*, and it was stored at 10 °C or 25 °C for 6 days, depending on the hurdle combination under investigation. After the habituation of each pathogen to the appropriate conditions, the *in vitro* digestion process was conducted as previously described. Samples were obtained at distinct time intervals during the oral, gastric and intestinal phase in order to characterize microbial growth dynamics. Serial decimal dilutions of the samples were prepared in 0.85% *w*/*v* NaCl solution and then inoculated on solid nutritional media. The inoculation of the plates was performed either by transferring three drops of 20 μL or by surface plating 100 μL of the appropriate dilution. For *S*. Typhimurium, TSA was used as a solid nutritional medium, whilst for *L. monocytogenes*, BHI agar was selected. The plates were incubated at 37 °C for 24–48 h and by the enumeration of colonies on the incubated plates, the cell density was determined. The number of biological replicates was two.

### 2.10. Modelling Microbial Kinetics

The model shown in Equations (1)–(3) was fitted to the experimental data obtained from the *in vitro* digestion process for both the gastric and the intestinal phases. This model describes a microbial inactivation curve consisting of a shoulder, a log-linear inactivation phase and a tail [20].

(1) $$\frac{\mathrm{dN}\left(t\right)}{\mathrm{dt}}=-\left(\frac{1}{1+C\left(t\right)}\right)\cdot r_{\max}\cdot \left(1-\frac{N_{\mathrm{res}}}{N\left(t\right)}\right)\cdot N\left(t\right)$$

(2) $$\frac{\mathrm{dC}\left(t\right)}{\mathrm{dt}}=-r_{\max}\cdot C\left(t\right)$$

(3) $$S_{L}=\frac{\ln\left(1+C_{0}\right)}{r_{\max}}$$

where N(t) (CFU) is the cell density at time t (min), r_max_ (1/min) is the maximum specific inactivation rate, N_res_ (CFU) is the residual population in the tail phase, C(t) (−) is a measure of the physiological state of the cells and S_L_ is the shoulder length (min). The initial conditions at t = 0 are denoted by N_0_ (CFU) for the initial cell density and C_0_ (−) for the initial physiological state of the cells. By omitting the first and/or the third factor of Equation (2), the model excluded the shoulder and/or the tailing phase when they were absent. The *lsqnonlin* routine of the Optimization Toolbox of MATLAB version R2015b (The Mathworks, Inc., Natick, MA, USA) was used for the minimization of the sum of squared errors to estimate the parameters of the mathematical model. Lastly, the parameter estimates were determined based on the Jacobian matrix [21] and the goodness of the model fit was based on the Mean Squared Error (MSE). For each dataset, the factors included in the model (shoulder/tail) were selected to obtain the lowest MSE with the fewest model parameters. When the estimated rate r_max_ < 0, it represents the maximum specific growth rate, and when r_max_ > 0, it represents the maximum specific inactivation rate.

### 2.11. Statistical Analysis

The analysis of variance (ANOVA) test was performed to determine whether there are any significant differences among means of logarithmically transformed viable counts, at a 95.0% confidence level (α = 0.05). The Tukey’s honestly significant difference (Tukey’s HSD) test was used for pairwise comparison of the ANOVA results. The analyses were performed using the anova1 routine of the Statistical Toolbox of MATLAB version R2018b. Test statistics were regarded as significant when *p* ≤ 0.05.

## 3. Results and Discussion

### 3.1. Assessment and Modelling of Growth Boundaries

Initially, the growth limits of *Salmonella* Typhimurium and *Listeria monocytogenes* were investigated in several different combinations of *a*_w_, pH and storage temperature of the developed FMS, as explained in Section 2.4. The reason for this was to select the appropriate hurdle combinations, to which each pathogen would be habituated prior to the *in vitro* digestion experiments.

**Figure 2:**
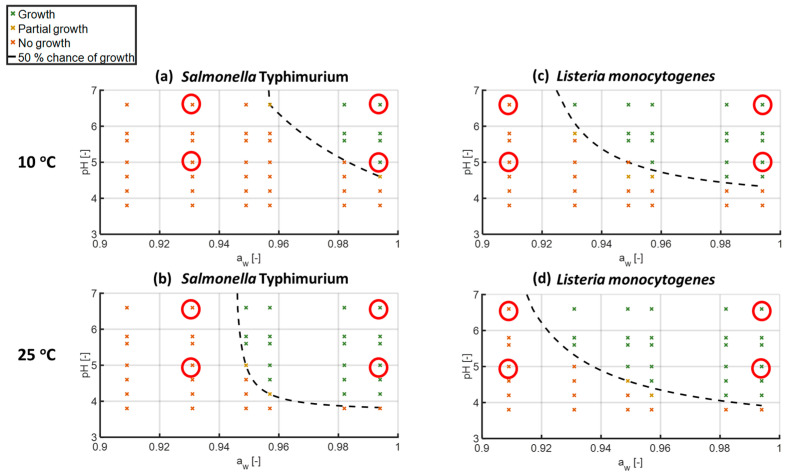
Growth/no growth interface of (**a**) *S*. Typhimurium at 10 °C, (**b**) *S*. Typhimurium at 25 °C, (**c**) *L. monocytogenes* at 10 °C and (**d**) *L. monocytogenes* at 25 °C, with respect to pH and *a*_w_ predicted by logistic regression model and compared with the data used to generate the model (**x** stands for growth, **x** stands for partial growth, **x** stands for no growth, the black dashed line for the 50% chance of growth and the red circles denote the hurdle combinations selected for the *in vitro* digestion experiments).

The results showed that for *S*. Typhimurium at 10 °C and 25 °C, the minimum *a*_w_ values for growth were 0.982 and 0.949, respectively, and the minimum pH values for growth were 5.0 and 4.2, respectively (Figure 2a,b). On the other hand, *L. monocytogenes* appeared more tolerant to the inimical environmental stresses, where the minimum *a*_w_ value for growth was 0.931 at both temperatures and the minimum pH values that permitted growth were 4.6 at 10 °C and 4.2 at 25 °C (Figure 2c,d). Similarly, previous studies have illustrated that *S*. Typhimurium required a higher minimum *a*_w_ value for growth (*a*_w_ 0.942) in a liquid broth at 25–35 °C than *L. monocytogenes* (*a*_w_ 0.900). Nevertheless, the minimum pH value for growth in the same conditions was higher for *L. monocytogenes* with a value of 4.45 compared with *S*. Typhimurium that required a minimum pH of 3.94 [22,23]. In general, comparing the boundaries of growth of the two bacteria, it is quite evident that *L. monocytogenes* exhibited broader margins of growth than *S*. Typhimurium, especially when the storage temperature was at 10 °C. These observations were quite expected, given that *L. monocytogenes* experienced less of an effect on the growth boundaries of *a*_w_ and pH due to the decreased temperature, being a psychrophile [24]. Additionally, for both microorganisms, there were a few examples of conditions close to the growth/no growth boundary where some replicates grew and others did not, being signified as combinations that had partial growth.

Remarkably, the results illustrated that the probability of growth may be altered dramatically by slight changes in *a*_w_ and pH, as has been previously demonstrated for *S*. Typhimurium, *L. monocytogenes* and *E. coli* [22,23,25]. In a similar manner, storage temperature played a crucial role in the growth limits of each pathogen, where, as expected, a higher temperature (25 °C) enabled more combinations of *a*_w_ and pH to permit growth. The latter indicates a synergistic effect of *a*_w_, pH and temperature on the growth limits of the two pathogens that is, in fact, observed in all of the investigated conditions. However, a previous study on the boundaries of growth of *S*. Typhimurium in tryptic soy broth exhibited a non-synergistic effect of the same hurdles when the *a*_w_ ranged between 0.990 and 0.955 [22]. A putative explanation for the inconsistency of these findings with our results can be that the current examined system, i.e., the FMS, was a viscous food model system rather than a liquid broth. In fact, several previous studies have illustrated differences between the growth limits of bacteria in solid and liquid media, indicating the significance of the medium’s state [23,26,27].

From the overall observation of the results, a total of eight different combinations of temperature (10 °C, 25 °C), pH (5.0, 6.6) and *a*_w_ (0.909 or 0.931, 0.994) were selected for the preparation and storage of the developed FMS. These combinations are clearly denoted in Figure 2 with the red circles. Out of these conditions, half belong to the growth region and the other half belong to the no-growth region, for both pathogens. The conditions representing the no-growth regions of each pathogen were chosen for their ability to inhibit growth but not to significantly inactivate the microbial population. *S*. Typhimurium expressed different and more narrow boundaries of growth than *L. monocytogenes*. Consequently, the selected combinations of hurdles for each bacterium differed, primarily in the lower value of *a*_w_, which was 0.931 for *S*. Typhimurium and 0.909 for *L. monocytogenes*.

### 3.2. Effect of the Hurdle Technology Application on the Survival of Salmonella Typhimurium and Listeria Monocytogenes

#### 3.2.1. *Salmonella* Typhimurium

1. General remarks

**Figure 3:**
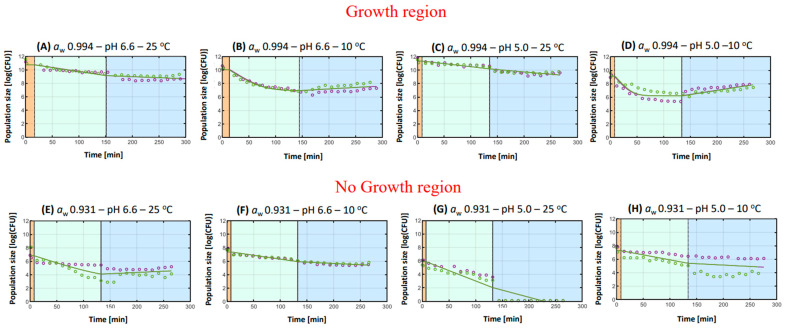
Effect of hurdle combinations of water activity (*a*_w_), pH and storage temperature on the microbial kinetics of *S*. Typhimurium during simulated digestion. The different phases of *in vitro* digestion, i.e., the oral phase, the gastric phase and the intestinal phase, are differentiated by a line and by the colors orange (left), light green (middle) and light blue (right), respectively. The experimental data are signified by the circles, while the lines represent the fit of the model for inactivation [20] to the data. The colors green and purple distinguish the two different replicates.

The microbial kinetics of *S*. Typhimurium are observed in Figure 3A–H, during each phase of simulated digestion. Table 1 illustrates the parameter estimates of the microbial kinetics of the pathogen obtained from the inactivation model [20], along with the Mean Squared Error (MSE) and the final log reduction. A general observation of the results revealed a linear trend during the intestinal phase for all eight experimental conditions selected and a more complex behavior during the gastric phase. More specifically, the results demonstrated inactivation of the pathogen during the gastric phase and less inactivation or even growth during the intestinal phase in all experimental conditions [16]. This phenomenon can be mainly attributed to the elevated sensitivity of *S*. Typhimurium toward the low values of gastric pH (2.5), along with its high tolerance against intestinal bile acids [10,28]. The great antimicrobial effects that gastric pH can infer on *S*. Typhimurium have been also previously demonstrated, where *S*. Typhimurium exhibited higher acid sensitivity than *L. monocytogenes* and *E. coli* when their microbial kinetics were compared under various pH values of simulated gastric fluid [28]. In addition, this finding is further corroborated by two of our previous studies, in which *S*. Typhimurium expressed similar behavior during the simulated digestion process when investigating the effects of gastric pH and intestinal bile acids or the effect of the food carrier properties on its survival during simulated digestion [16,29].

With respect to the different hurdle combinations, they were distinguished in growth and no growth environmental conditions, with the hurdle combinations with an *a*_w_ of 0.994 permitting the growth of *S*. Typhimurium in the FMS (growth region) and those with an *a*_w_ of 0.931 inhibiting the growth of the pathogen (no growth region) (Figure 3). In fact, previous research has revealed that the minimum *a*_w_ for the growth of *S*. Typhimurium is 0.94, further corroborating our findings [30]. Hence, as illustrated from the results, even though the inoculum level in all conditions was 10^9^ CFU, in the beginning of digestion (t = 0 min), the initial population of *S*. Typhimurium had increased at conditions A–D and reduced at conditions E–H. For instance, in condition C, the initial population of *S*. Typhimurium was 11.347 log(CFU) (Figure 3C), while in condition F, the initial bacterial cell density was significantly lower with a value of 5.887 log(CFU) (Figure 3F).

2. Hurdles during *in vitro* digestion

The results revealed that the hurdle combination with the lowest inactivation was condition D (*a*_w_ 0.994–pH 5.0–10 °C), with the final log reduction (throughout the whole duration of simulated digestion) being the lowest (1.303 log(CFU)) and with a residual population at the end of the gastric phase (6.202 log(CFU)) (Figure 3D). In contrast, the hurdle combination that was related to the highest inactivation of *S*. Typhimurium during digestion was condition F (*a*_w_ 0.931–pH 5.0–25 °C), which inferred the highest final log reduction in the pathogen with a value of 6.629 log(CFU) (Figure 3F). As such, condition D was associated with increased bacterial stress tolerance, which is a phenomenon that could be attributed to the “stress-hardening” phenomenon triggered by the previous exposure of pathogen to a low *a*_w_ and low pH in the FMS that eventually enabled its survival in the acidic environment of the simulated stomach [7]. To obtain a better understanding of how the different hurdle combinations in the FMS affected the survival patterns of *S*. Typhimurium during digestion, an elaborate discussion is included, focusing each time on a single hurdle’s impact.

##### Hurdle of *a*_w_

At the conditions where the pH and the storage temperature of the FMSs were similar, but the *a*_w_ differed, a significant finding that was observed was that a low *a*_w_ in the FMS (0.931) was often associated with increased inactivation of *S*. Typhimurium during simulated gastric digestion, while an *a*_w_ of 0.994 inferred the tolerance of the pathogen against gastric acidity. For instance, observing the final log reduction values of conditions C (Figure 3C, *a*_w_ 0.994–pH 5.0–25 °C) and G (Figure 3G, *a*_w_ 0.931–pH 5.0–25 °C), it is quite clear that microbial inactivation was significantly higher at condition G (gastric r_max_ +0.031 1/min and final log reduction 6.629 log(CFU)) than at condition C (gastric r_max_ +0.009 1/min and final log reduction 2.067 log(CFU)). As such, our results revealed that in the case of acidic stress (gastric acidity), a previous exposure to a low *a*_w_ was able to induce the acid tolerance of *S*. Typhimurium. Previous research concerning the acid responses of *Salmonella* after exposure to low *a*_w_ foods is limited, yet the prolonged survival of *Salmonella* in low *a*_w_ values has been observed to induce the tolerance of the pathogen against heat [31,32,33].

Another important finding was that the effect of *a*_w_ on the acid stress responses of *S*. Typhimurium during gastric digestion was greatly dependent on the FMS’s storage temperature, indicating a synergistic or additive effect. More specifically, when the *a*_w_ was 0.994, a storage temperature of 10 °C resulted in the extensive inactivation of *S*. Typhimurium during the gastric phase (Figure 3B,D), where the highest gastric r_max_ values were observed, i.e., +0.052 1/min for condition B (*a*_w_ 0.994–pH 6.6–10 °C) and +0.095 1/min for condition D (*a*_w_ 0.994–pH 5.0–10 °C). On the contrary, when the *a*_w_ of the FMS was low and at the no-growth region of *S*. Typhimurium (0.931), a storage temperature of 10 °C was associated with a more acid-tolerant microbial behavior during simulated gastric digestion (Figure 3F,H). For example, the values of gastric r_max_ for conditions F and H were significantly lower (+0.015 and +0.012 1/min, respectively) when compared to conditions E and G (+0.031 and 0.026 1/min, respectively), in which the storage temperature was higher (25 °C). *Salmonella* is a mesophilic bacterium with an optimal temperature range of 30–45 °C and with the minimum temperature for growth being 5.2 °C [30,34]. Thereby, the habituation of *S*. Typhimurium to hurdle combinations at the growth region (*a*_w_ 0.994) with a storage temperature as low as 10 °C presumably inflicted cell damage to the pathogen that caused, in turn, its significant inactivation during the acidic gastric phase. In contrast, when *S*. Typhimurium was previously exposed to the same temperature at 10 °C but at the no-growth region (*a*_w_ 0.931), the simultaneous application of multiple sublethal stresses triggered its “stress hardening”, resulting in the pathogen’s elevated acid tolerance against gastric acidity.

##### Hurdle of pH

At the conditions where the *a*_w_ and the storage temperature of the FMSs were the same but the pH was different, the results showed a diverged microbial behavior of *S*. Typhimurium between the conditions that were at the growth region and those that were at the no-growth region, indicating the influence of *a*_w_ exposure on the antimicrobial effects of gastric pH.

When the *a*_w_ was at the growth region of the pathogen (0.994), a low pH value in the FMS resulted in the increased tolerance of *S*. Typhimurium against gastric acidity when compared to a higher pH value. For example, the highest inactivation of the pathogen was observed at condition B (*a*_w_ 0.994–pH 6.6–10 °C) when the pH was high (pH 6.6) (Figure 3B) with a final log reduction of 2.497 log(CFU). Yet, when the pH of the FMS was lower (Figure 3D, *a*_w_ 0.994–pH 5.0–10 °C), *S*. Typhimurium demonstrated a significantly tolerant behavior against gastric acidity with a final log reduction of 1.303 log(CFU), which was, in fact, the lowest log reduction reported in this study’s results. In fact, acid habituation of *S*. Typhimurium has been previously associated with increased microbial tolerance toward several sources of otherwise lethal stress, such as heat, acidity and osmotic stress, with the literature suggesting that a pH range of 4.0–5.0 may infer excessive resistance to this bacterium [9,35,36].

On the other hand, when the *a*_w_ was at the no-growth region of *S*. Typhimurium (0.931), a low pH value in the FMS (pH 5.0) caused the extensive inactivation of the pathogen during simulated digestion, while a higher pH value (6.6) was associated with a more tolerant microbial behavior. For instance, in condition G (Figure 3G, *a*_w_ 0.931–pH 5.0–25 °C), a pH of 5.0 contributed to the highest final reduction in the pathogen, along with a significantly higher gastric r_max_ (+0.031 1/min), when compared to the r_max_ value of +0.026 1/min obtained for condition E (Figure 3E, *a*_w_ 0.931–pH 6.6–25 °C). The association of the higher pH values with increased acid tolerance is also illustrated by the limited final log reduction values, e.g., 2.251 log(CFU) for condition E and 1.962 log(CFU) for condition F, as well as by the presence of a residual population that was often observed. The exposure of *Salmonella* to mild acidity may activate the ATR of the pathogen that can, in turn, enable its survival at otherwise lethal conditions [8]. The latter, in combination with the growth-inhibiting levels of *a*_w_ in the FMS, could, in fact, induce the ATR of *S*. Typhimurium. The combined effect of low *a*_w_ with other sublethal stresses on microbial survival has also been previously demonstrated when *Salmonella* Typhimurium expressed a greater acid tolerance in SGF after its previous exposure to a low *a*_w_ and mildly acidic dry-cured meat product at 25 °C [9].

##### Hurdle of Storage Temperature

When focusing on the effect of storage temperature, the results showed that when the FMS was stored at a higher temperature (25 °C), *S*. Typhimurium was greatly inactivated during simulated digestion, whilst a storage temperature of 10 °C was associated with limited inactivation of the pathogen. This finding was rather expected, since bacteria have the capacity to synthesize cold-shock proteins to hinder the lethal effects of cold shock [37]. For instance, *S*. Typhimurium has been reported to produce the cold-shock protein B (CspB), as a response to temperature decreases below 24 °C [38]. In addition, it has been revealed that CspB can widely promote oxidative, pH, osmotic, starvation and ethanol stress tolerance, playing, as such, an active role in bacterial cross-protection [39]. Hence, when comparing the microbial kinetics of *S*. Typhimurium after habituation to the growth-inhibiting conditions G and H, the final log reduction and the gastric r_max_ were higher in condition G (Figure 3G, *a*_w_ 0.931–pH 5.0–25 °C), where the storage temperature was 25 °C (6.629 log(CFU), +0.031 1/min, respectively), than in condition H (Figure 3H, *a*_w_ 0.931–pH 5.0–10 °C), where the storage temperature was 10 °C (2.408 log(CFU), +0.015 1/min, respectively). In fact, condition H corresponds to the harshest environmental conditions for microbial survival; nonetheless, *S*. Typhimurium exhibited a relatively limited final log reduction in its population, as well as the second lowest gastric r_max_ value, indicating an adaptive tolerance of the pathogen to the gastricidal effects of the simulated stomach. Interestingly, the different levels of *a*_w_ and pH did not exhibit a considerable impact on the general effect of storage temperature on the behavior of *S*. Typhimurium during simulated digestion. Thereby, when at the growth region, a habituation of the pathogen to conditions with a low storage temperature resulted as well in increased microbial tolerance, with an apparent residual population and proliferation during the simulated intestinal phase. For instance, the habituation of *S*. Typhimurium to conditions B (Figure 3B, *a*_w_ 0.994–pH 6.6–10 °C) and D (Figure 3D, *a*_w_ 0.994–pH 5.0–10 °C) manifested a similar residual population of 6.931 log(CFU) and 6.202 log(CFU), respectively, as well as proliferation during intestinal digestion with an r_max_ of −0.004 1/min and of −0.012 1/min, respectively.

#### 3.2.2. *Listeria monocytogenes*

1. General remarks

**Figure 4:**
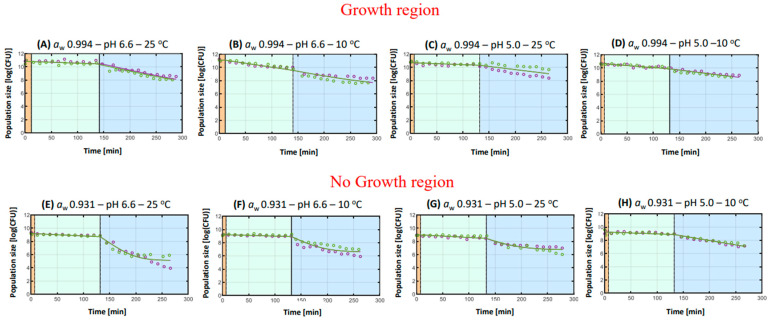
Effect of hurdle combinations of water activity (*a*_w_), pH and storage temperature on the microbial kinetics of *L. monocytogenes* during simulated digestion. The different phases of *in vitro* digestion, i.e., the oral phase, the gastric phase and the intestinal phase, are differentiated by a line and by the colors orange (left), light green (middle) and light blue (right), respectively. The experimental data are signified by the circles, while the lines represent the fit of the inactivation model [20] to the data. The colors green and purple distinguish the two different replicates.

Figure 4A–H demonstrate the microbial kinetics of *L. monocytogenes* during each phase of the *in vitro* digestion. Table 2 shows the parameter estimates of the microbial kinetics of the pathogen obtained from the inactivation model [20], along with the Mean Squared Error (MSE) and the final log reduction. A general trend that was observed was a more complex behavior during the intestinal phase, whereas the gastric phase was mainly dictated by linearity. In addition, the results revealed a high acid tolerance of *L. monocytogenes* during the acidic gastric phase (pH 3.0) and sensitivity of the pathogen to the bactericidal effects of intestinal bile acids [16]. A previous study has illustrated that *L. monocytogenes* was characterized by a greater acid tolerance than *S*. Typhimurium when they were both subjected to an acid challenge test with lactic acid (pH 3.5) [36]. In addition, during the simulated intestinal phase, *L. monocytogenes* was mainly inactivated due to the bactericidal effects of bile acids, and a residual population was often observed. These findings are in concordance with previous studies, where *L. monocytogenes* expressed a similar behavior when the effect of gastric pH and intestinal bile acids or the effect of food-buffering capacity and food composition on its survival were under investigation during *in vitro* digestion [16,29]. In fact, the apparent sensitivity of *L. monocytogenes* to bile acids could be explained by the pathogen’s previous exposure to gastric acidity that can infer a subsequent susceptibility to the bactericidal properties of bile acids [16]. In addition, previous findings have exhibited that bile sensitivity could be pH-dependent when *L. monocytogenes* strains manifested a decreased survival after their exposure to bile acids at a pH value of 5.5, which is a case similar to this study [40].

Concerning the different experimental conditions, they were distinguished in growth and no growth conditions, as previously explained. Consequently, conditions A–D were in the growth region, where, as illustrated in Figure 4A–D, the initial microbial cell densities of *L. monocytogenes* in the beginning of digestion and after its habituation to the different environmental conditions were higher than 10^9^ CFU, which was the FMS’s inoculum level (e.g., at condition A the N_0_ was 10.808 log(CFU)). On the other hand, conditions E–H characterize the hurdle combinations that did not permit the growth of *L. monocytogenes* (Figure 4E–H); hence, the initial population levels of the pathogen did not increase significantly. For instance, as observed at condition E, the N_0_ was 9.172 log(CFU).

2. Hurdles during *in vitro* digestion

The hurdle combination that led to the highest subsequent inactivation of *L. monocytogenes* during simulated digestion was condition E (*a*_w_ 0.909–pH 6.6–25 °C), where the final log reduction in the pathogen was estimated at 4.032 log(CFU) and the intestinal r_max_ was +0.055 1/min, rendering the highest estimated values among the different experimental conditions (Figure 4E). On the other side, the lowest inactivation was obtained at hurdle combination C (*a*_w_ 0.994–pH 5.0–25 °C), where the final log reduction in the pathogen was significantly lower than the rest of the cases, with a value of 1.657 log(CFU), indicating an acquired microbial tolerance of *L. monocytogenes* to the bactericidal effects of intestinal bile acids (Figure 4C). Indeed, *L. monocytogenes* has exhibited the capacity to express adaptive stress responses via the process of “stress hardening” after being exposed to sublethal levels of acidity (pH 5.0–5.5), that can, in turn, enhance its capacity to survive its passage through the human GIT [41,42]. The interpretation of the results is organized in the same manner as for *S*. Typhimurium, focusing each time on the effects of one individual hurdle.

##### Hurdle of *a*_w_

When the hurdles of pH and storage temperature were alike, but the *a*_w_ levels differed, the results illustrated that in most cases, a lower *a*_w_ was responsible for the subsequent elevated inactivation of *L. monocytogenes* during intestinal digestion, while a higher *a*_w_ was often associated with microbial tolerance. For instance, after the pathogen was habituated to condition A (Figure 4A, *a*_w_ 0.994–pH 6.6–25 °C), its population exhibited a significantly lower final log reduction (2.728 log(CFU)) and a significantly lower intestinal r_max_ (+0.018 1/min) during simulated digestion when compared to condition E (*a*_w_ 0.909–pH 6.6–25 °C) (Figure 4E), that, as previously explained, exhibited the highest values of final log reduction and intestinal r_max_. Overcoming osmotic stress is considered an energy-depleting process for bacteria, since great amounts of metabolic energy are required to accumulate compatible solutes intracellularly in order to limit water loss [43,44]. Thereby, the continuous energy depletion due to the exposure to a series of subsequent stresses, e.g., osmotic stress, acid stress in the stomach, and bile acids toxicity in the intestine, could explain the increased inactivation of *L. monocytogenes* after its habituation to sublethal levels of *a*_w_. Interestingly, the lowest inactivation of *L. monocytogenes* that was observed at condition C (*a*_w_ 0.994–pH 5.0–25 °C) was at the growth region of the pathogen (Figure 4C), and it could indicate a plausible microbial tolerance to the antimicrobial effects of digestion.

##### Hurdle of pH

Turning now to the impact of pH on microbial behavior, the results demonstrated that when the hurdles of *a*_w_ and storage temperature were similar, a lower pH value was associated with a higher microbial tolerance compared with a higher pH. More specifically, when the hurdle combinations permitted the growth of *L. monocytogenes* (*a*_w_ 0.994), the final log reduction in the pathogen was higher when the pH was 6.6. Nonetheless, when the pH was 6.6, a residual population was apparent in all experimental conditions. For instance, in condition A (Figure 4A, *a*_w_ 0.994–pH 6.6–25 °C), the final log reduction in *L. monocytogenes* at the end of simulated digestion was 2.728 log(CFU), while at condition C (Figure 4C, *a*_w_ 0.994–pH 5.0–25 °C), it was 1.657 log(CFU), which was the lowest final log reduction among all cases. Likewise, when comparing conditions F (Figure 4F, *a*_w_ 0.909–pH 6.6–10 °C) and H (Figure 4H, *a*_w_ 0.909–pH 5.0–10 °C), it is quite evident that the final log reduction is significantly higher in condition F (2.573 log(CFU)) than in condition H (2.147 log(CFU), along with the intestinal r_max_ (+0.041 1/min in condition F and +0.013 1/min in condition H), yet a tail is observed with a value of 6.584 log(CFU) (condition F). These findings can be attributed to the occurrence of cross-protection, where the low pH of the FMS stressed the cells of *L. monocytogenes*, leading to its subsequent hardening on the exposure to the sublethal stresses of gastrointestinal digestion. In fact, the so-called “stress-hardening” phenomenon can be associated with the induction of ATR after the previous exposure of the pathogen to acidic conditions [7,45]. The effects of cross-protection and “stress hardening” on *L. monocytogenes* have been previously illustrated, where the pathogen exhibited an increased acid tolerance to gastric acidity (pH 2.0) after it had been previously habituated to a pH of 5.5 and 6.6 for 15 days [46]. Moreover, a recent previous study revealed that the habituation of *L. monocytogenes* to a pH of 5.5 or 6.0 induced a greater ATR that a pH of 6.5 [47], readily confirming the current findings.

##### Hurdle of Storage Temperature

When focusing on the storage temperature, a high value (25 °C) during the microbial habituation to the FMS was related with the increased tolerance of *L. monocytogenes* against the bactericidal effects of simulated digestion, whereas a low storage temperature (10 °C) inferred the pathogen’s increased sensitivity and, as a result, its greater inactivation during digestion. For instance, the final log reduction in *L. monocytogenes* after habituation to condition A (Figure 4A, *a*_w_ 0.994–pH 6.6–25 °C) was 2.728 log(CFU), whilst in condition B (Figure 4B, *a*_w_ 0.994–pH 6.6–10 °C), where the storage temperature was at 10 °C, the final log reduction was significantly higher and had a value of 3.303 log(CFU). In addition, at condition A, a significantly lower gastric r_max_ was observed (+0.003 1/min) in comparison with the gastric r_max_ value in condition B (+0.012 1/min). Furthermore, comparing conditions G (Figure 4G, *a*_w_ 0.909–pH 5.0–25 °C) and H (Figure 4H, *a*_w_ 0.909–pH 5.0–10 °C), even though the final log reduction values are similar, it is quite evident that when the storage temperature of the FMS was at 25 °C, *L. monocytogenes* exhibited a residual population during intestinal digestion (6.374 log(CFU)), indicating obtained microbial tolerance to the antimicrobial effects of the intestinal bile acids. An explanation for the apparent lack of stress tolerance of *L. monocytogenes* after being habituated to conditions where the environmental temperature was 10 °C could be that given the slower growth rate of the pathogen at this low temperature, a lesser fraction of its population would be at the late stationary phase at the end of storage time. Indeed, the exposure of *L. monocytogenes* to low temperatures first leads to its cell arrest (acclimation) and then to its adaptation, where the microbial population is able to proliferate but at a slower rate [48]. Consequently, this could readily characterize this microbial population as considerably more sensitive to the subsequent gastrointestinal stresses, since previous studies have clearly demonstrated that the stationary phase cells are more stress tolerant than the exponential phase ones [49].

## 4. Conclusions

Hurdle technology is a vastly applied method for food preservation, chiefly depending on the combined effects of multiple sublethal stresses that eliminate potential microbial threats in food products or keep them under control. The exposure of microorganisms to these stresses can sensitize them or harden them toward other types of subsequent stressful environments, such as the human GIT. The survival of pathogens in the GIT may be detrimental for a host’s health, as it can lead to infection and disease. The present results revealed that both *S*. Typhimurium and *L. monocytogenes* survived their transit through the simulated GIT with an increased microbial tolerance often being observed for both pathogens after their habituation to certain hurdle combinations.

**Figure 5:**
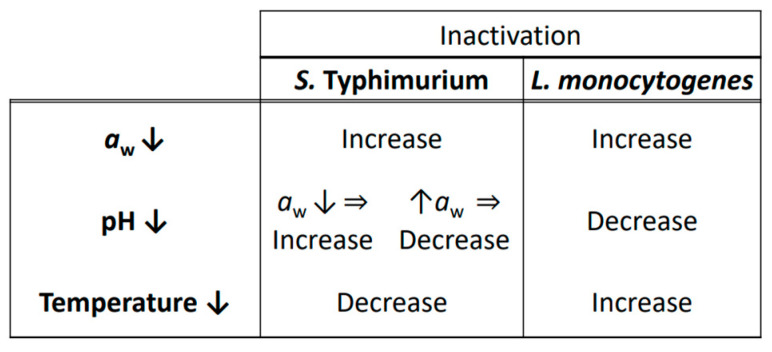
Overview of microbial responses during *in vitro* digestion, focusing on the impact of a single hurdle.

Taking a closer look at the influence of each individual hurdle applied (Figure 5), this research showed that the habituation of the pathogens to environmental conditions that did not permit their growth (low *a*_w_) sensitized them toward the antimicrobial effects of gastrointestinal digestion. In addition, for *S*. Typhimurium: (i) the hurdle of a low pH was linked with increased sensitivity to gastric acidity when the *a*_w_ was low (higher microbial inactivation) but with increased tolerance when the *a*_w_ was high (lower microbial inactivation) and (ii) a low storage temperature caused the increased inactivation of the pathogen, which was due to adaptive stress responses. On the other hand, for *L. monocytogenes*, (i) its habituation to low pH values was related to its decreased inactivation and hence to a more tolerant behavior and (ii) a low storage temperature of the FMS led to its increased sensitivity against intestinal bile acids, as indicated by the increased microbial inactivation. Lastly, in some cases, a synergistic/additive effect was exhibited between certain hurdles, e.g., *a*_w_ and pH for *S*. Typhimurium. It is clear that the selection of hurdle combinations for optimal food preservation is a complex matter that might lead to unforeseen outcomes for microbial survival. In fact, stress-tolerant cells may have an increased chance to overcome the gastrointestinal barriers of the human digestive system, posing a major threat for public health. Therefore, further research is required to convey a better view of the cross-protection effects that hurdle technology may infer on bacterial pathogens during their gastrointestinal transit. The future work should draw more attention to (i) the assessment of different types of hurdles and/or different hurdle combinations that are also commonly used in the food industry, shedding more light on their potential synergistic effects, and to (ii) the implementation of an *in vitro* digestion system that takes into account the complexity of the real GIT with its antimicrobial barriers, such as the resident gut microbiota.

## Figures and Tables

**Table 1:** Kinetic parameters estimates of the inactivation model [20], both for the simulated gastric phase and the simulated intestinal phase, obtained for the cells of *S.* Typhimurium during the *in vitro* digestion of the different food model systems ^1,2^.

		Gastric Phase					Intestinal Phase		
Experimental Condition		r_max_ (1/min)		N_res_ [log(CFU)]		MSE	r_max_ (1/min)		Final Log Reduction [log(CFU) ± SD]
			95% Confidence Interval					95% Confidence Interval	
**A**	***a*_w_ 0.994–pH 6.6–25 °C**	+0.012 ^a^	[+0.009, +0.015]	-	-	0.107	+0.003 ^a^	[+0.001, +0.006]	2.023 ± 0.167 ^a^
**B**	***a*_w_ 0.994–pH 6.6–10 °C**	+0.052 ^b^	[+0.041, +0.062]	6.931 ^a^	[6.716, 7.147]	0.128	−0.004 ^b^	[−0.003, −0.001]	2.497 ± 0.199 ^b^
**C**	***a*_w_ 0.994–pH 5.0–25 °C**	+0.009 ^c^	[+0.007, +0.011]	-	-	0.070	+0.007 ^c^	[+0.005, +0.009]	2.067 ± 0.132 ^a^
**D**	***a*_w_ 0.994–pH 5.0–10 °C**	+0.095 ^d^	[+0.072, +0.019]	6.202 ^a^	[5.933, 6.467]	0.285	−0.012 ^d^	[−0.018, −0.007]	1.303 ± 0.265 ^c^
**E**	***a*_w_ 0.931–pH 6.6–25 °C**	+0.026 ^g^	[+0.015, +0.037]	3.288 ^b^	[0.236, 6.341]	0.495	−0.004 ^b^	[−0.012, +0.004]	2.251 ± 0.287 ^b^
**F**	***a*_w_ 0.931–pH 6.6–10 °C**	+0.012 ^a^	[+0.010, +0.013]	-	-	0.045	+0.004 ^e^	[+0.002, +0.005]	1.962 ± 0.103 ^a^
**G**	***a*_w_ 0.931–pH 5.0–25 °C**	+0.031 ^f^	[+0.024, +0.038]	-	-	0.754	+0.021 ^f^	[+0.014, +0.028]	6.629 ± 0.423 ^d^
**H**	***a*_w_ 0.931–pH 5.0–10 °C**	+0.015 ^e^	[+0.006, +0.023]	-	-	0.988	+0.004 ^e^	[+0.003, +0.011]	2.408 ± 0.492 ^b^

^1^ The superscript lowercase letters indicate the differences obtained by ANOVA between the same kinetic parameters for each experimental condition. Parameter estimates that do not share the same letter are significantly different (*p* < 0.05). ^2^ The r_max_ estimates indicate growth when r_max_< 0, or inactivation when r_max_ > 0.

**Table 2:** Kinetic parameters estimates of the inactivation model [20], both for the simulated gastric phase and the simulated intestinal phase, obtained for the cells of *Listeria monocytogenes* during the *in vitro* digestion of the different food model systems ^1,2^.

Experimental Condition		Gastric Phase		Intestinal Phase					Final Log Reduction [log(CFU) ± SD]
		r_max_ (1/min)		r_max_ (1/min)		N_res_ [log(CFU)]		MSE	
			95% Confidence Interval		95% Confidence Interval		95% Confidence Interval		
**A**	***a*_w_ 0.994–pH 6.6–25 °C**	+0.003 ^a^	[+0.002, +0.003]	+0.018 ^a^	[+0.013, +0.024]	6.698 ^a^	[3.388, 10.009]	0.066	2.728 ± 0.146 ^a^
**B**	***a*_w_ 0.994–pH 6.6–10 °C**	+0.012 ^b^	[+0.009, +0.015]	+0.016 ^b^	[+0.005, +0.027]	7.000 ^a^	[4.550, 9.450]	0.120	3.303 ± 0.225 ^b^
**C**	***a*_w_ 0.994–pH 5.0–25 °C**	+0.003 ^a^	[+0.002, +0.004]	+0.010 ^c^	[+0.005, +0.001]	-		0.167	1.657 ± 0.177 ^c^
**D**	***a*_w_ 0.994–pH 5.0–10 °C**	+0.006 ^c^	[+0.006, +0.007]	+0.010 ^c^	[+0.009, +0.011]	-		0.030	1.939 ± 0.077 ^d^
**E**	***a*_w_ 0.909–pH 6.6–25 °C**	+0.004 ^d^	[+0.001, +0.007]	+0.055 ^g^	[+0.045, +0.065]	5.115 ^b^	[4.783, 5.448]	0.150	4.032 ± 0.189 ^g^
**F**	***a*_w_ 0.909–pH 6.6–10 °C**	+0.002 ^e^	[+0.001,+0.004]	+0.041 ^f^	[+0.028, +0.053]	6.584 ^a^	[6.238, 6.931]	0.123	2.573 ± 0.158 ^f^
**G**	***a*_w_ 0.909–pH 5.0–25 °C**	+0.004 ^d^	[+0.002,+0.006]	+0.033 ^e^	[+0.017, +0.049]	6.374 ^a^	[6.459, 7.009]	0.070	2.216 ± 0.131 ^e^
**H**	***a*_w_ 0.909–pH 5.0–10 °C**	+0.003 ^a^	[+0.002,+0.004]	+0.013 ^d^	[+0.012, +0.015]	-		0.030	2.147 ± 0.075 ^e^

^1^ The superscript lowercase letters indicate the differences obtained by ANOVA between the same kinetic parameters for each experimental condition. Parameter estimates that do not share the same letter are significantly different (*p* < 0.05). ^2^ The r_max_ estimates indicate growth when r_max_ < 0, or inactivation when r_max_ > 0.

## Data Availability

The data presented in this study are available on request from the corresponding author.
